# A preclinical study on the influence of linkers in [^68^Ga]Ga-NOTA-X-RM26 radiotracers for PET imaging of GRPR expression

**DOI:** 10.1186/s13550-025-01301-y

**Published:** 2025-08-07

**Authors:** Esther Olaniran Håkansson, Ivan V. Zelepukin, Karim Obeid, Athanasios Bitzios, Ekaterina Bezverkhniaia, Amulya Sunkara, Ulrika Rosenström, Anna Orlova, Luke R. Odell, Panagiotis Kanellopoulos

**Affiliations:** 1 Department of Medicinal Chemistry, Uppsala University, Uppsala, 751 23 Sweden; 2 Department of Immunology, Genetics and Pathology, Uppsala University, Uppsala, 751 83 Sweden; 3 Science for Life Laboratory, Uppsala University, Uppsala, 752 37 Sweden

**Keywords:** Radiopharmaceuticals, GRPR, Bombesin analogue, Antagonist, Prostate cancer, Structure-properties relationship

## Abstract

**Background:**

Gastrin-releasing peptide receptor (GRPR) is overexpressed in several cancers, including prostate and breast, making it an attractive target for radiopharmaceutical development. Studies on GRPR-targeting radioligands highlight the critical role of the spacer region between the GRPR-recognition motif and radiolabeled moiety, which can significantly influence peptide pharmacokinetics and pharmacodynamics. Herein, we investigated the impact of structurally restricted spacers on the performance of RM26-based radioligands.

**Results:**

Three novel radioligands were designed to each bear a NOTA chelator via different spacers composed of *N*-acetyl-lysine followed by either *o*-ethyltoluene (*o*ET), *o*-methylanisole (*o*MA), or *m*-methylanisole (*m*MA) motifs. The peptides were successfully labeled with Ga-68, achieving high radiochemical yield, purity, and molar activity. The resulting [^68^Ga]-labeled peptides demonstrated high and GRPR-specific binding to prostate cancer PC-3 cells, antagonistic behavior, and the IC_50_ values to GRPR were in the single-digit nanomolar range. Biodistribution studies at 2 h post-injection in PC-3 xenograft-bearing mice revealed high, GRPR-mediated tumor uptake for all three radioligands. In addition, high hepatobiliary excretion with elevated uptake in the liver and the gastrointestinal tract and pronounced pancreatic uptake were observed.

**Conclusions:**

Among the three radioligands, the peptide bearing the *N*-acetyl-lysine-*o*ET spacer exhibited the fastest background clearance and better PET imaging of prostate cancer xenografts. The incorporation of conformationally restricted spacers is a promising strategy for developing tracers with high GRPR binding and good imaging properties, but further optimization is necessary to reduce uptake in healthy tissues.

**Supplementary Information:**

The online version contains supplementary material available at 10.1186/s13550-025-01301-y.

## Background

Early detection and precise staging of tumor lesions are important for effective cancer management. Among tomographic imaging techniques, positron emission tomography (PET) plays a key role in the detection of metastases, guiding personalized therapeutic strategies, and evaluating drug treatment response. PET is a highly sensitive, non-invasive imaging modality with a spatial resolution of several mm, equal or surpassing the resolution of single-photon emission computed tomography (SPECT) [1]. It enables real-time visualization and quantification of radiotracer uptake in malignant tissues, providing valuable insights into tumor metabolism, receptor expression, and lesion heterogeneity [2]. However, this technique requires the development of targeted radiotracers with high specificity for cancer tissues and minimal uptake in healthy organs to ensure accurate and safe tumor imaging.

The gastrin releasing peptide receptor (GRPR) is a promising target for PET imaging due to its overexpression in multiple malignancies, including prostate cancer, breast cancer, gastrointestinal (GI) stromal tumors, and small-cell lung cancer [3, 4, 5, 6]. Since GRPR expression in normal tissues is largely restricted to the pancreas and gastrointestinal organs, targeting this receptor enables early detection of cancer lesions with high specificity. Moreover, GRPR expression in these healthy tissues is at least one order of magnitude lower than in clinical tumor samples [7]. For breast cancer, it has been shown that a high level of GRPR expression in a primary tumor is a valuable tool for predicting metastasis probability and is strongly associated with estrogen receptor (ER) expression [8]. In addition, GRPR-targeted PET imaging is particularly advantageous in prostate cancer cases where prostate-specific membrane antigen (PSMA) expression is heterogeneous or absent, providing a complementary targeting approach [9].

RM26 is a GRPR antagonist that exhibits high specificity for GRPR and has therefore shown promise in PET imaging [10]. While RM26 has demonstrated improved pharmacokinetic properties, further optimizations are needed to improve tumor retention and minimize background signal, especially in non-target tissues such as the kidneys and liver [11]. Radioligands generally consist of three key components: the targeting molecule, a linker, and a radiometal chelator. The incorporation of different linkers can drastically influence the pharmacokinetic and pharmacodynamic properties of the radioligand, a concept often referred to as “linkerology” [12]. The linker influences the overall topology and lipophilicity of the radiotracer, which in turn affects receptor affinity, tissue distribution, and clearance. For example, Bombesin [2 − 14] derivatives incorporating hydrophilic linkers exhibit increased affinity for GRPR but also demonstrate higher reabsorption in kidneys [13]. Therefore, the choice of linker remains a crucial tool to fine-tune the overall properties of a radioligand.

A family of RM26 targeting radiopeptides with flexible hydrophilic polyethelyneglycol (PEG) linkers has been evaluated as GRPR radioligands for prostate cancer imaging [14, 15, 16]. However, biodistribution was largely unaffected by varying the PEG-linker chain length, while increased hydrophilicity decreased affinity to GRPR target [17]. This highlights the need to explore alternative linker moieties, which incorporate properties such as rigidity and varied polarity. For instance, RM1 and RM2 GRPR radioligands with short rigid linkers (Gly-4-aminobenzoyl and piperidine, respectively) demonstrated potent preclinical and clinical results for PET imaging [18, 19, 20]. The precise effects of different linkers remain challenging to predict, highlighting the need for systematic investigations.

In this study, we designed a novel family of RM26-based GRPR targeting peptides having partially restricted linkers with an *α*-acetyl-lysine and one of the restricted turns in their structure: *o*-ethyltoluene, *o*-methylanisole, or *m*-methylanisole. We examined the impacts of conformational rigidity and linker polarity on GRPR binding, pharmacokinetics, and PET imaging in mice. Notably, incorporation of these linkers did not change the antagonistic behavior of ^68^Ga-labelled radiopeptides, and affinity to GRPR remained in the nanomolar range. The analogue with an *o*-ethyltoluene turn demonstrated lower off-target uptake in liver and lungs, with clear imaging of human prostate cancer PC-3 xenografts. Our study demonstrates the successful design of potent GRPR radioantagonists featuring non-linear, conformationally restricted structures, and holds strong potential to significantly expand the range of diagnostic and therapeutic radioligands available for PET imaging of prostate cancer.

## Materials and methods

### Materials

Ethanol, sodium acetate, hydrochloric acid, citric acid, ethylenediaminetetraacetic acid (EDTA), trifluoroacetic acid (TFA), acetonitrile, RPMI 1640 cell medium with L-glutamine, bovine serum albumin (BSA) were purchased from Sigma-Aldrich (USA). Penicillin–streptomycin solution was obtained from MP Biomedicals (USA). Fetal bovine serum (FBS) and trypsin-0.25% EDTA solution were purchased from Gibco (USA). Ketamine hydrochloride was purchased from Pfizer (USA). Xylazine hydrochloride was obtained from Bayer (Germany). Ga-68 was acquired by elution of an IGG100 Ge-68/Ga-68 generator (Eckert & Ziegler, Germany). Metal-free water was prepared from Milli-Q water treated with Chelex 100 resin (Sigma-Aldrich, USA).

### Synthesis

Analytical HPLC/ESI-MS was performed using electrospray ionization (ESI) and a C18 column (50 × 3.0 mm, 2.6 μm particle size, 100 Å pore size) with CH_3_CN/0.05% aqueous HCOOH as mobile phase at a flow rate of 1.5 mL/min. Preparative reversed-phase high-performance liquid chromatography (RP-HPLC) was performed by UV-trigged (214 nm) fraction collection with a Glison HPLC system using a Machery-nagel NUCLEODUR C18 HTec column (21 × 125 mm, particle size 5 μm) and H_2_O/CH_3_CN with 0.1% v/v TFA as mobile phase at a flow rate of 25 mL/min. High-resolution mass spectra (HRMS) were determined on a mass spectrometer equipped with an ESI source and a time-of-flight (TOF) mass analyzer. All chemicals and solvents were purchased from Sigma Aldrich, Fisher Scientific, and VWR, and used without further purification.

### Resin-bound RM26

The synthesis of RM26 (D-Phe-Gln-Trp-Ala-Val-Gly-His-Sta-Leu-NH_2_) was carried out by standard Fmoc solid phase peptide synthesis on Fmoc Rink Amide 4-Methylbenzhydrylamine (MBHA) resin (loading 0.69 mmol/g) in a 6 mL disposable syringe, similarly as described before [21]. Shortly, sequential couplings were performed in DMF using PyBOP (4 equiv.) as coupling reagent and DIPEA (7 equiv.) as a base. All couplings were carried out for at least two hours, and Fmoc-deprotection was performed using 20% piperidine in DMF followed by washing with DMF.

### General procedure for synthesis of NOTA-linker-RM26 conjugates

**Fig. 1:**
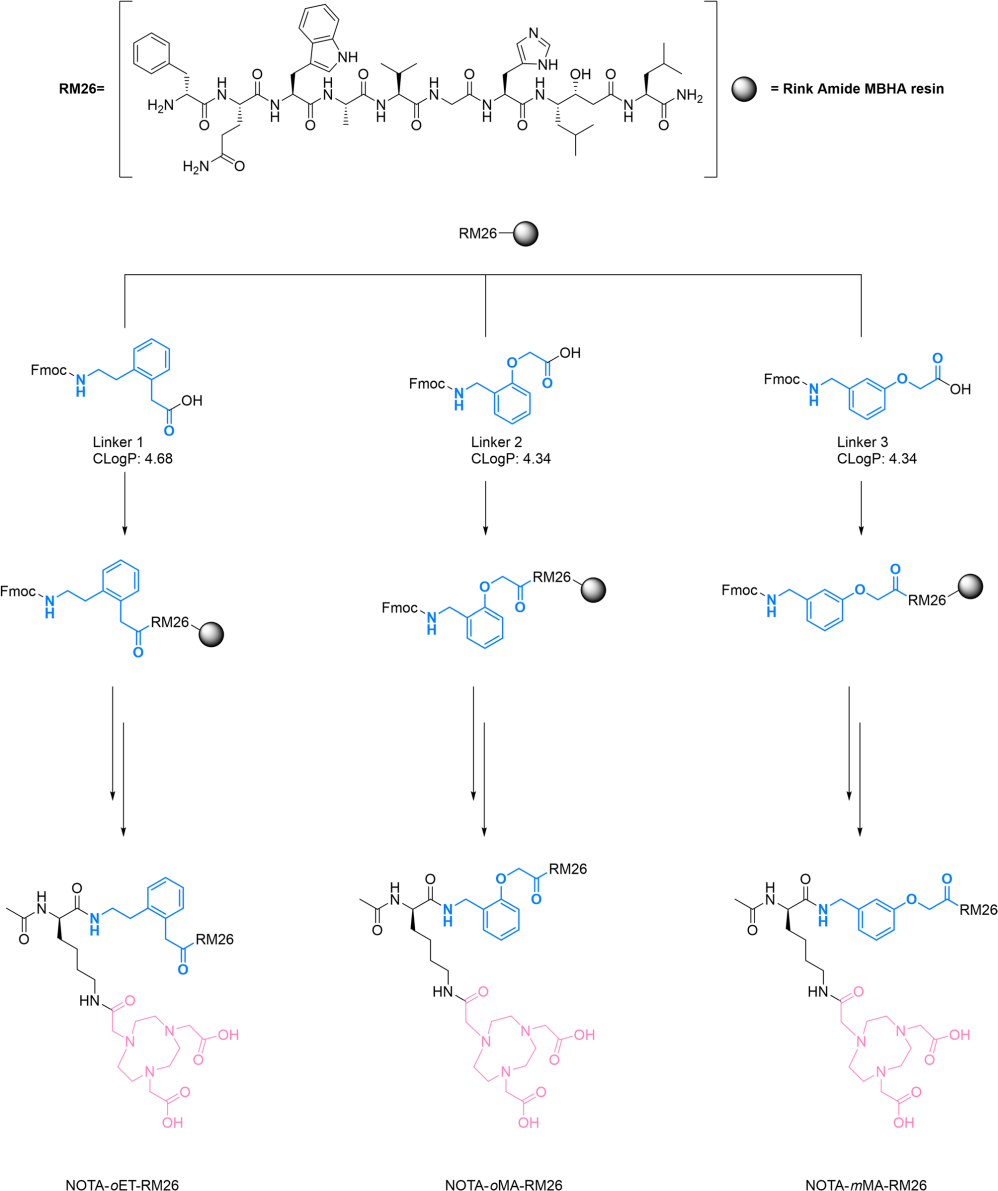
Synthesis and chemical structure of NOTA-*o*ET-RM26, NOTA-*o*MA-RM26, and NOTA-*m*MA-RM26 compounds, where blue represents the rigid linkers, pink the NOTA chelator, and RM26 the GRPR targeting peptide. CLogP values were calculated using ChemDraw 23.1.2

The synthesis of the peptide conjugates proceeded according to Fig. 1. Resin-bound RM 26 (60 mg, 15.5 µmol) was transferred to a 3 mL syringe with a filter and swollen in DCM for 30 min. All couplings were performed for 4 h using PyBOP and DIPEA in DMF, and Fmoc was removed using 20% piperidine in DMF. After every coupling and Fmoc-removal, the resin was subsequently washed with DMF. First, the Fmoc-protected non-natural amino acid linkers **1–3** (77.6 µmol, 5 equiv.) and Fmoc-Lys(Alloc)-OH (62 µmol, 4 equiv.) were coupled sequentially. Then, alloc deprotection was carried out in the presence of PhSiH_3_ (0.31 mmol, 20 equiv.) and Pd(PPh_3_)_4_ (4.7 µmol, 0.3 equiv.) in DCM for 3 h. Finally, NOTA was coupled to the resin using the above-stated conditions. After removal of Fmoc on lysine, the *N*-terminal was acetylated by treating the resin with acetic anhydride (0.78 mmol, 50 equiv.) in DMF and piperidine (0.78 mmol, 50 equiv.) for 30 min. Cleavage and global deprotection were accomplished by subjecting the resin to TFA: H_2_O: thioanisole: triethylsilane (87:5:6:3) for 3 h. The resulting solution was concentrated under a stream of nitrogen and precipitated in diethyl ether. The white precipitate was washed twice with cold diethyl ether. The crude was purified using reversed-phase high-performance liquid chromatography (RP-HPLC) with 0.1% TFA in water and 0.1% TFA in acetonitrile as mobile phases. Pure fractions were lyophilized to obtain the final peptides as white solids, which were characterized by HRMS and analyzed by analytical RP-HPLC-MS.

### NOTA-*o*ET-RM26

The title compound was synthesized according to the general procedure using 2-(2-(2-((((9*H*-fluoren-9-yl)methoxy)carbonyl)amino)ethyl)phenyl)acetic acid (Linker 1, Fig. 1) (32 mg, 77.6 mmol, 5 equiv.) as the non-natural amino acid linker. Yield 3.6 mg, 12%. The purity of the peptide was confirmed by LC-MS (Figure S1,S2). Peptide identity was confirmed by HRMS (ESI/TOF) m/z: [M + H]^+^ calculated for C_85_H_125_N_20_O_19_ 1729.9430; found 1729.9358 (Figure S3).

### NOTA-*o*MA-RM26

The title compound was synthesized according to the general procedure using 2-(2-(((((9*H*-fluoren-9-yl)methoxy)carbonyl)amino)methyl)phenoxy)acetic acid (Linker 2, Fig. 1) (31 mg, 77.4 mmol, 5 equiv.) as the non-natural amino acid linker. Yield 3.9 mg, 14%. The purity of the peptide was confirmed by LC-MS (Figure S4,S5). Peptide identity was confirmed by HRMS (ESI/TOF) m/z: [M + H]^+^ calculated for C_84_H_123_N_20_O_20_ 1731.9223; found 1731.9171 (Figure S6).

### NOTA-*m*MA-RM26

The title compound was synthesized according to the general procedure using 2-(3-(((((9*H*-fluoren-9-yl)methoxy)carbonyl)amino)methyl)phenoxy)acetic acid (31 mg, 77.4 mmol, 5 equiv.) as the non-natural amino acid linker. Yield 4.3 mg, 15%. The purity of the peptide was confirmed by LC-MS (Figure S7,S8). Peptide identity was confirmed by HRMS (ESI/TOF) m/z: [M + H]^+^ calculated for C_84_H_123_N_20_O_20_ 1731.9223; found 1731.9023 (Figure S9).

### Radiolabeling and purification

Ga-68 was eluted from an IGG100 Ge-68/Ga-68 generator using a 0.1 M metal-free HCl solution. Then, 10 nmol of peptides in Milli-Q water were dissolved in 250 µL of 1 M sodium acetate (pH 4.5) and mixed with 120 MBq of ^68^Ga eluate. The solution was heated for 10 min at 75 °C and cooled down to room temperature.

The radiolabeling yield was measured by instant thin layer chromatography (iTLC). 1 µL of the peptide solution was dropped onto a glass microfiber paper impregnated with silica gel (Agilent Technologies, USA), and the paper was developed with a 0.2 M citric acid solution. After paper impregnation, ^68^Ga distribution across the strip was measured by a Cyclone Plus phosphorimager (Perkin Elmer, USA).

If the radiolabeling yield was lower than 90%, peptides were purified by Sep-Pak tC18 cartridges. The column was pretreated with 1 mL of ethanol and 1 mL of metal-free water, and the peptide was loaded. Then, five 200 µL fractions were collected by eluting 1 mL of a 50% ethanol aqueous solution. The peptide was collected from the second and third fractions, and the isolated yield was measured by ^68^Ga activity.

### Radiolabeling purity

Radiolabeling purity was measured by reverse-phase radio-HPLC analysis using a LaPrep Sigma system equipped with an LP1100 pump (Hitachi, Japan), a UV-detector 40D LWL (Knauer, Germany), a flow scan radioactivity detector with an FC-3300 NaI/PMT probe (Echert&Ziegler, Germany) and a Rheodyne 7725i sample injector fitted with a 20 µL loop (Thermo Scientific, USA). HPLC conditions were as follows: a Luna C18 column (5 μm, 100 Å, 150 × 4.6 mm from Phenomenex, Denmark), 1 mL/min flow rate, and UV detection at 220 nm. The following solution gradients were applied during HPLC (A– 0.1% TFA in water; B– 0.1% TFA in acetonitrile): 0 min– 95% A and 5% B; 20 min– 40% A and 60% B.

Complex stability was measured by incubation of 1 µM protein in either PBS buffer (pH 7.4) or 1 mM EDTA solution in PBS for 1 h at room temperature. The release of ^68^Ga was quantified by iTLC analysis.

Octanol-water distribution coefficient (LogD) was determined by adding 10 pmol of radiopeptide to an Eppendorf tube containing 500 µL of phosphate-buffered saline (PBS) and 500 µL of n-octanol. Each tube was vortexed for 2 min and centrifuged at 6000 g for 5 min. Three 100 µL fractions were collected from each phase, and their radioactivity was measured.

### Cell studies

PC-3 cells of human prostatic adenocarcinoma were obtained from the American Type Culture Collection (ATCC). Cells were cultured in RPMI 1640 medium containing L-glutamine and supplemented with 10% FBS and penicillin-streptomycin solution, under a humidified atmosphere at 5% CO_2_ and 37 °C.

To measure the specificity of peptide binding, cells were seeded in 6-well plates at a density of 10^6^ cells per well and grown overnight. The medium was then removed, and the cells were washed with 1 mL of PBS and incubated with 1 mL of a 1 nM radiopeptide solution in complete medium. To block GRPR receptors on the cells’ surface, they were treated with 1 µM of non-radiolabeled NOTA-PEG_2_-RM26 solution in complete medium and incubated for 15 min at room temperature before the addition of the radiopeptide. The cells were incubated at 37 °C for 1 h, the supernatant was discarded, the cells were washed twice with 1 mL of PBS, and collected by incubation with trypsin-0.25% EDTA solution. Radioactivity of the cells was measured using a Wizard 2480 automatic gamma counter (PerkinElmer, USA). The measurements were performed in triplicates (*n* = 3).

To measure cellular processing of radiopeptides, PC-3 cells were seeded in 35-mm Petri dishes at a density of 8·10^5^ cells per dish and grown overnight. Then, the cells were washed with 1 mL of PBS and incubated with 1 mL of 1 nM radiolabeled peptide solution in complete medium at 37 °C. At 0.5, 1, 2, or 3 h, the medium was removed, the cells were washed with cold PBS, and incubated with 600 µL of acid wash (0.2 M glycine, 0.15 M NaCl, 4 M urea, pH 2) for 5 min over ice. Then, supernatant was collected, and the cells were washed with 600 µL of the same acid buffer to collect the radioligand detached from the cell membrane. Cells were further incubated with 600 µL of 1 M NaOH for 15 min at 37 °C to separately collect internalized peptide. The activity content of the samples was measured by a Wizard 2480 gamma counter, and the data were normalized to the maximum detected cell-associated activity. The measurements were performed in triplicates (*n* = 3).

### Competitive binding

Metalation of the peptides with ^nat^Ga was performed following a similar protocol used for radiolabeling. In short, 30 nmol of the respective peptide was mixed with 10 µL of 9 mM GaCl_3_, 150 µL of 0.1 M HCl, and 250 µL of 1 M sodium acetate (pH 4.5). The mixture was then incubated at 70 °C for 60 min.

PC-3 cells were seeded in 12-well plates at 10^5^ cells per well density and incubated overnight. Then, the media was aspirated, and cells were washed with 500 µL of cold PBS buffer, containing 1% w/v bovine serum albumin (PBS/BSA buffer). Then the cells were treated with 350 µL of cold PBS/BSA buffer, 50 µL of the metalated ligand at different concentrations (from 0 to 1000 nM), and 100 µL of [^125^I]I-Tyr^4^-bombesin (PerkinElmer, Waltham, MA, USA) in PBS/1% BSA buffer, at 24.6 fmol/well concentration. Cells were incubated for 5 h at 4°C to reach equilibrium of binding. After that, the supernatant was removed, cells were washed with cold PBS buffer containing 1% BSA, and collected using 1 mL of 1 M NaOH heated to 37 °C. The radioactivity was measured by a Wizard 2480 gamma counter (PerkinElmer, Waltham, MA, USA). The experiments were performed in triplicates (*n* = 3) and the data were fitted to the curves with a nonlinear regression model in GraphPad Prism 7.

### Animals

Female BALB/c nu/nu mice of 18–22 g weight were used in the study. All procedures with animals were conducted in accordance with the EU Directive 2010/63/EU for animal experiments and Swedish national legislation concerning the protection of laboratory animals. The experiments were approved by the ethics committee for animal research in Uppsala (Sweden) with permit number 5.8.18–00473/2021. Animals were housed in individually ventilated cages, under a 12-h light/dark cycle with free access to food and water.

For tumor inoculation, 8·10^6^ PC-3 cells in 100 µL PBS were injected subcutaneously into the right hind leg of mice. Mouse weight and tumor size were measured every 2–3 days, and tumor volume was estimated by ellipsoid approximation. A predefined humane endpoint of the experiment was either a 15% weight loss or a tumor volume of 1000 mm^3^, neither of which was reached.

### Biodistribution analysis

Four weeks after tumor cell implantation, mice were randomized and divided into 6 groups (4 animals / group): 3 of them received treatment with ^68^Ga-radiolabeled peptides, while the other 3 received the same peptides in addition to 5 nmol of non-labeled NOTA-PEG_2_-RM26 peptide for GRPR-blocking. Peptides were injected intravenously via the tail veins at a dose of 50 pmol (700 kBq radioactivity dose).

At 2 h post injection, mice were sacrificed by overdose of Ketamine/Xylazine anesthesia, and blood was collected by heart puncture. Then, liver, spleen, lungs, pancreas, kidneys, small intestine, stomach, tail, femur bones, tumor and muscle from the opposite legs were collected, weighed, and radioactivity was measured using a Wizard 2480 gamma counter. Data are presented as percentages of injected activity per gram of tissue (%IA/g).

### PET/CT imaging

For PET/CT imaging, animals bearing PC-3 xenografts were used. The animals received a single intravenous injection of the corresponding ^68^Ga-radiolabeled peptide (100 pmol, 1.2 MBq) and 2 h post injection they were euthanized using CO_2_. For image acquisition, Nano PET 3T (PET/MRI) and nanoScan (SPECT/CT) (Mediso Medical Imaging Systems, Budapest, Hungary) tomographs were used. Reconstruction of the PET scans was conducted using Nucline nanoScan 3.04.014.0000 software. CT data were reconstructed using Filter Back Projection in Nucline 2.03 Software (Mediso Medical Imaging Systems Ltd., Budapest, Hungary). For their fusion, Nucline 2.03 Software was used, and PET images are presented as maximum intensity projections (MIP) with an equivalent color scale.

### Statistical analysis

All the experiments were performed at least in triplicate. Data are presented as mean ± SD. Statistical differences were analysed by two-tailed Welch’s t-test, since data dispersion greatly varied between the groups. *P* values less than 0.05, 0.01, and 0.001 are denoted as *, **, and ***, respectively.

## Results

### Targeting peptide design and synthesis

We designed three GRPR-targeting analogues based on NOTA-PEG_2_-RM26 [22] to investigate how the linker between the NOTA chelator and the RM26 targeting motif influences peptide interaction with the target both in vitro and in vivo. Each linker included one of the moderately hydrophobic and restricted turns: *o*-ethyltoluene (*o*ET ), *o*-methylanisole (*o*MA ), *m*-methylanisole (*m*MA) linked through an *α*-acetyl-lysine bridge to the chelator. The peptides were synthesized by manual Fmoc/tBu solid phase peptide synthesis (SPPS) on rink amide MBHA resin (Fig. 1) and were treated with TFA: H_2_O: thioanisole: triethylsilane to achieve global deprotection after assembly of the desired peptide sequence. After purification and lyophilization, the peptides were obtained as TFA salts in 12%, 14%, and 15% yield for NOTA-*o*ET-RM26, NOTA-*o*MA-RM26, and NOTA-*m*MA-RM26, respectively. The purity of the peptides was confirmed by LC-MS and characterized by high resolution mass spectrometry (HRMS) as depicted in Figures S1-S9.

### Ga-68 radiolabeling

The novel peptides were then labeled with Ga-68 radionuclide via NOTA chelation. The radiochemical yields (RCY, Table 1) for the *o*MA and *m*MA peptides were higher than 90%, therefore, these compounds did not require purification after labeling for in vitro experiments. For further in vivo experiments, all three compounds were purified by solid-phase extraction, and the radiochemical purity of the used compounds was higher than 99% (Figure S10). Incubation of labeled peptides at 37 °C for 1 h in a 1000-fold excess of EDTA induced only a low 0.4–2.5% additional release of the radionuclide compared to incubation in PBS buffer (Table 1). This confirms the stability of radiolabeling, which should persist in vivo.

The chemical and radiochemical purity of the compounds were accessed by HPLC analysis. Non-labeled peptides had chemical purity higher than 98%, with a single peak for the peptides (Figure S11). After labelling, radio-HPLC demonstrated that the radiochemical purity was 90%, 80%, and 88% for *o*ET, *o*MA, and *m*MA peptides, respectively (Figure S11). The amount of free Ga-68 or attached to a released chelator was lower than 5% (not decay corrected). The remaining activity eluted close to the major peptide peak and is most likely due to radiolysis products or measurement’s artifacts.

When their LogD values were calculated, the three novel radioligands had clearly higher values than the [^68^Ga]Ga-NOTA-PEG2-RM26, which was the most hydrophilic. Amongst them, *o*MA was the most lipophilic, followed by *o*ET and the most hydrophilic was *m*MA (Table 1).

**Table 1:** Radiochemical yields for ^68^Ga-labeled compounds, their stability over incubation at 37 °C for 1 h in PBS and PBS with 1000-fold molar excess of EDTA and their logd values. Data are presented as mean ± sd

Compound	RCY (%) (*n* = 4)	Stability in PBS (*n* = 3)	Stability in PBS with EDTA (*n* = 3)	LogD (*n* = 3)
[^68^Ga]Ga-NOTA-*o*ET-RM26	88.5 ± 2.5	95 ± 2.9	92.6 ± 4.7	-0.97 ± 0.01
[^68^Ga]Ga-NOTA-*o*MA-RM26	95 ± 1	91.7 ± 5.1	91.3 ± 5.2	-0.86 ± 0.02
[^68^Ga]Ga-NOTA-*m*MA-RM26	92.4 ± 3.1	92.8 ± 3.3	90.6 ± 4.1	-1.21 ± 0.03
[^68^Ga]Ga-NOTA-PEG2-RM26				-1.43 ± 0.01

### *In vitro* experiments

**Fig. 2:**
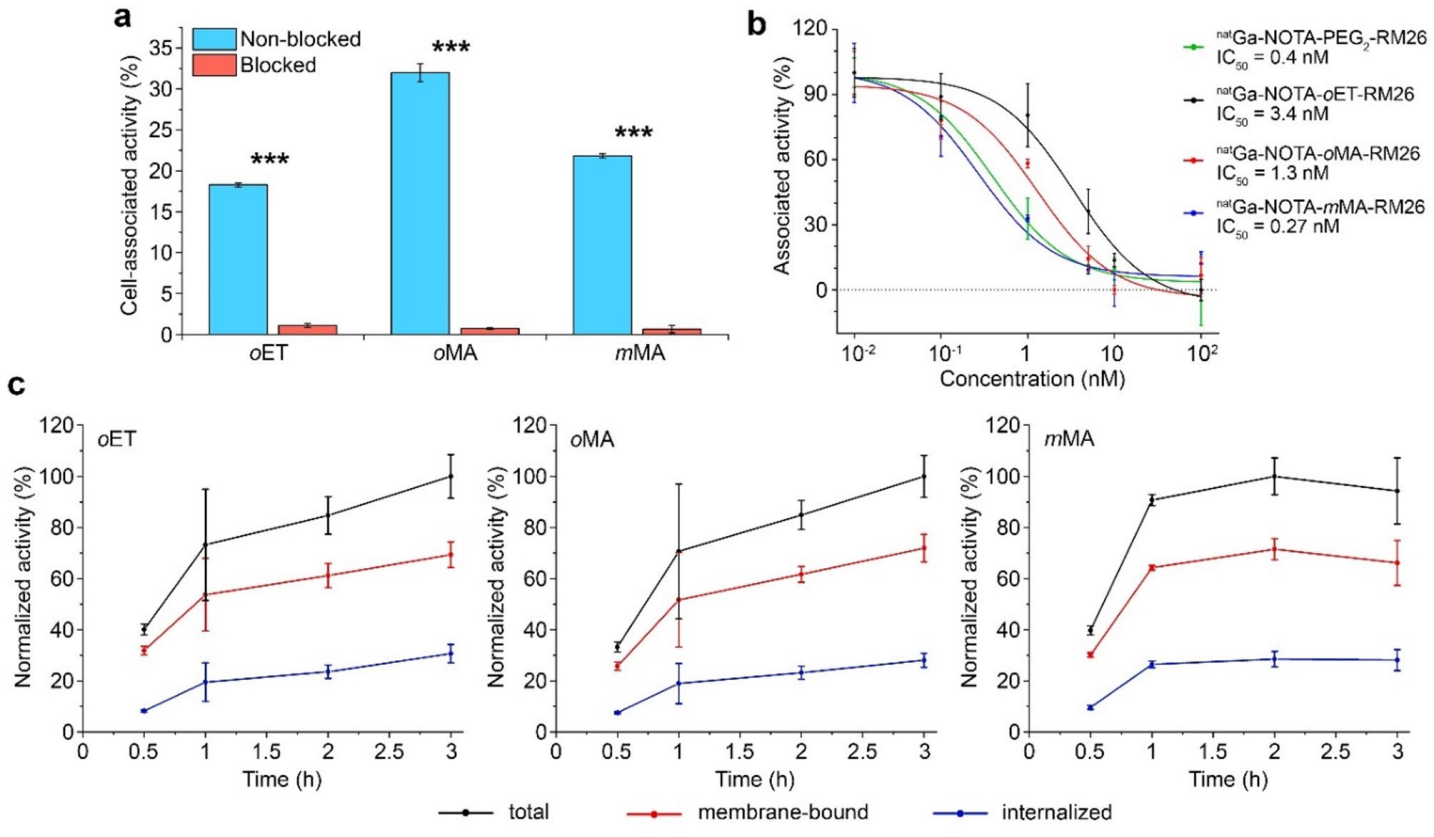
(**a**) Specificity tests of ^68^Ga-labeled *o*ET, *o*MA and *m*MA peptides binding to PC-3 cells in absence or presence of receptor blockade by excess of non-radiolabeled NOTA-PEG_2_-RM26. (**b**) Competition binding curves against [^125^I]I-Tyr^4^-bombesin ^nat^Ga-labeled oET, oMA and mMA peptides in comparison with ^nat^Ga-NOTA-PEG_2_-RM26. (**c**) Cellular processing of ^68^Ga-labeled *o*ET, *o*MA and *m*MA peptides by PC-3 cells over time. In (**a-c**) data are presented as mean ± SD. *** - *P* < 0.001, Welch’s t-test. All experiments were done in triplicates (*n* = 3)

For in vitro experiments, we chose the human PC-3 prostate cancer cell line, which has a high expression of GRPR at the level of 4·10^5^ receptors per cell [23]. All three ^68^Ga-labeled peptides demonstrated specific GRPR binding to PC-3 cells, as saturation of receptors with an excess of non-labeled NOTA-PEG_2_-RM26 peptide significantly decreased the amount of cell-associated activity. The specific binding of the *o*MA peptide was at least 1.5-fold higher than for other compounds (Fig. 2a). The specificity (i.e., ratio of specific to non-specific binding) was 16.6, 45, and 33.5 for *o*ET, *o*MA, and *m*MA peptides, respectively.

To evaluate the difference in affinity between designed radioligands, all compounds were loaded with natural gallium, and their IC_50_ values were determined against [^125^I]I-Tyr^4^-bombesin using PC-3 cells. We compared all peptides with ^nat^Ga-NOTA-PEG_2_-RM26, a potent analogue bearing a short hydrophilic diethylene glycol linker [22]. All the metalated compounds had IC_50_ values in the single-digit nanomolar range (Fig. 2b).

The cellular processing of ^68^Ga-labeled GRPR binding peptides was assessed in PC-3 cells over 3 h (Fig. 2c). The amount of cell-associated peptide increased within the first hour of incubation and then either slowly increased (*o*ET, *o*MA) or plateaued (*m*MA). This corresponds to the rapid receptor saturation and the establishment of an association-dissociation equilibrium in the medium. Peptide internalization proceeded slowly, with 28–30% internalization fraction of cell-associated activity for all compounds, indicating that linker alteration did not influence the internalization process. In addition, the internalized amount was significantly lower on ice, confirming that the low uptake level was not due to measurement error (Figure S12).

### Biodistribution and tumor targeting

**Fig. 3:**
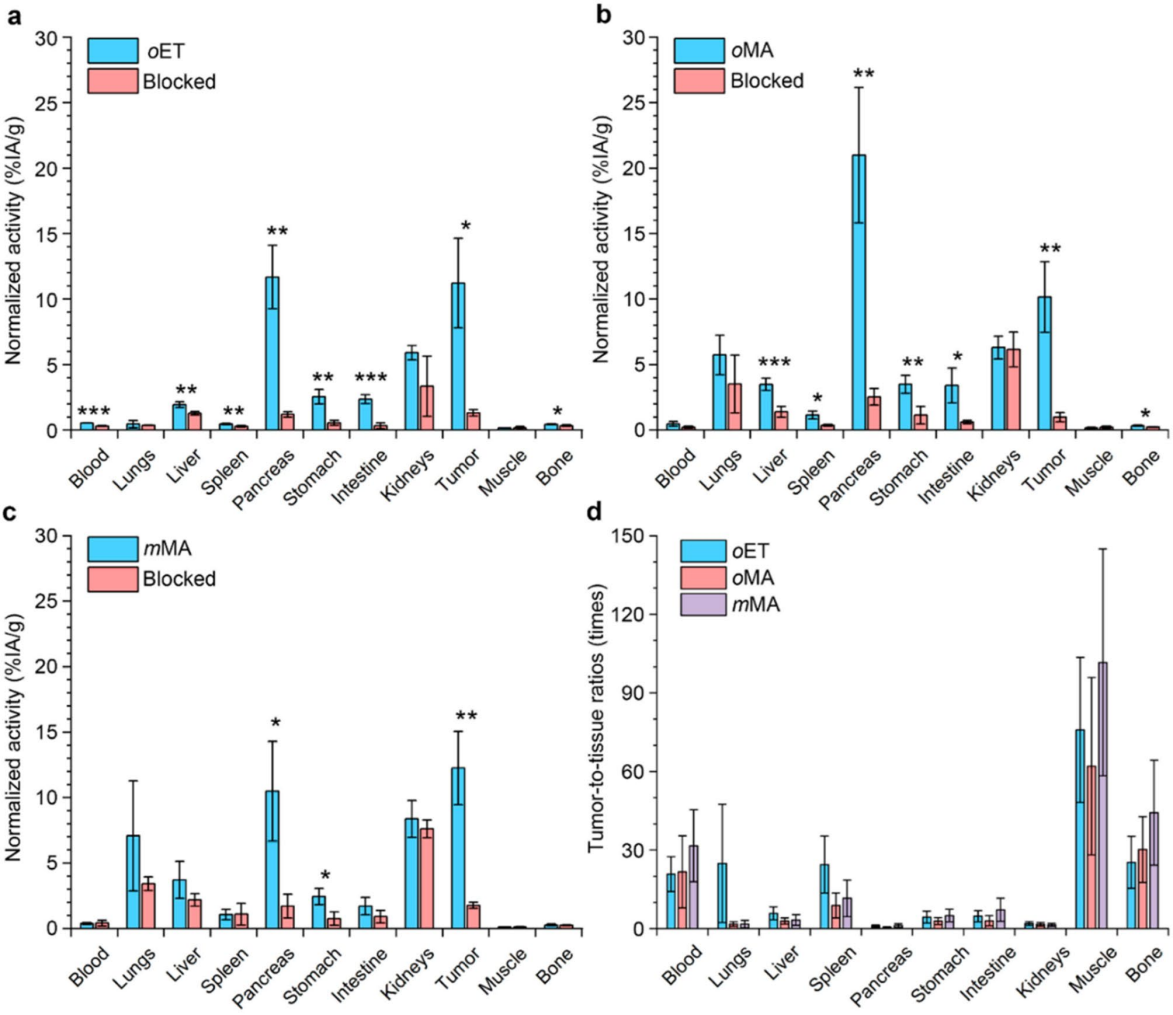
(**a-c**) Biodistribution and specificity of ^68^Ga-labeled *o*ET (**a**), *o*MA (**b**), *m*MA (**c**) peptides. Blockade was induced by co-injection of 100-fold excess of NOTA-PEG_2_-RM26 GRPR-binding peptide. (**d**) Tumor-to-tissues ratios of the activity accumulation for *o*ET, *o*MA, *m*MA peptides. Data are presented as mean ± SD. * - *P* < 0.05, ** - *P* < 0.01, *** - *P* < 0.001, Welch’s t-test. For all groups *n* = 4

The biodistribution of ^68^Ga-labeled conjugates was assessed in BALB/c nu/nu mice bearing human PC-3 xenografts (Fig. 3, raw data in Tables S1-3). The peptides demonstrated rapid whole-body clearance and excretion with 16.3%, 19.8%, and 16.5% of the injected activity (IA) detected in mice at this time point for *o*ET, *o*MA, and *m*MA, respectively. This demonstrates the high stability of ^68^Ga-labeled conjugates in vivo, which did not undergo radionuclide exchange with blood proteins.

The highest activity uptake was observed in the pancreas and in tumors, which correlates well with high GRPR expression in these tissues. The *o*MA peptide demonstrated at least a 1.7-fold higher accumulation in the pancreas, compared to other peptides. Tumor activity uptake was similar among the three compounds (11.2, 10.2, and 12.3%IA/g for *o*ET, *o*MA, and *m*MA analogues, respectively). The activity uptake was GRPR-specific and could be blocked by co-injection of a 100-fold dose of non-labelled NOTA-PEG_2_-RM26 peptide. Furthermore, receptor blocking decreased radionuclide accumulation in the pancreas and tumor by an order of magnitude. Blocking also decreased peptide uptake in the stomach and small intestine, which have moderate GRPR expression in mammals [24].

The moderate activity uptake in the kidney (6–8% IA/g) was non-GRPR specific and indicates participation of renal clearance in peptide elimination. Additionally, we observed moderate peptide accumulation (≤ 7.1% IA/g) in the liver and lungs, likely due to off-target interactions with other receptors or plasma proteins. Interestingly, the *o*ET peptide had the lowest activity uptake in these tissues. All other non-GRPR-expressing organs demonstrated activity uptake below 2% IA/g.

All 3 compounds demonstrated similar levels of activity uptake in tumors. Figure 3 shows the tumor-to-organ ratios for three peptides. The best tumor-to-liver ratio was found for *o*ET peptide, due to low activity accumulation in the liver. The tumor-to-muscle (62–102), tumor-to-blood (21–32) and tumor-to-bone ratios (25–44) indicated that high contrast image could be obtained already 2 h pi.

### PET/CT imaging

**Fig. 4:**
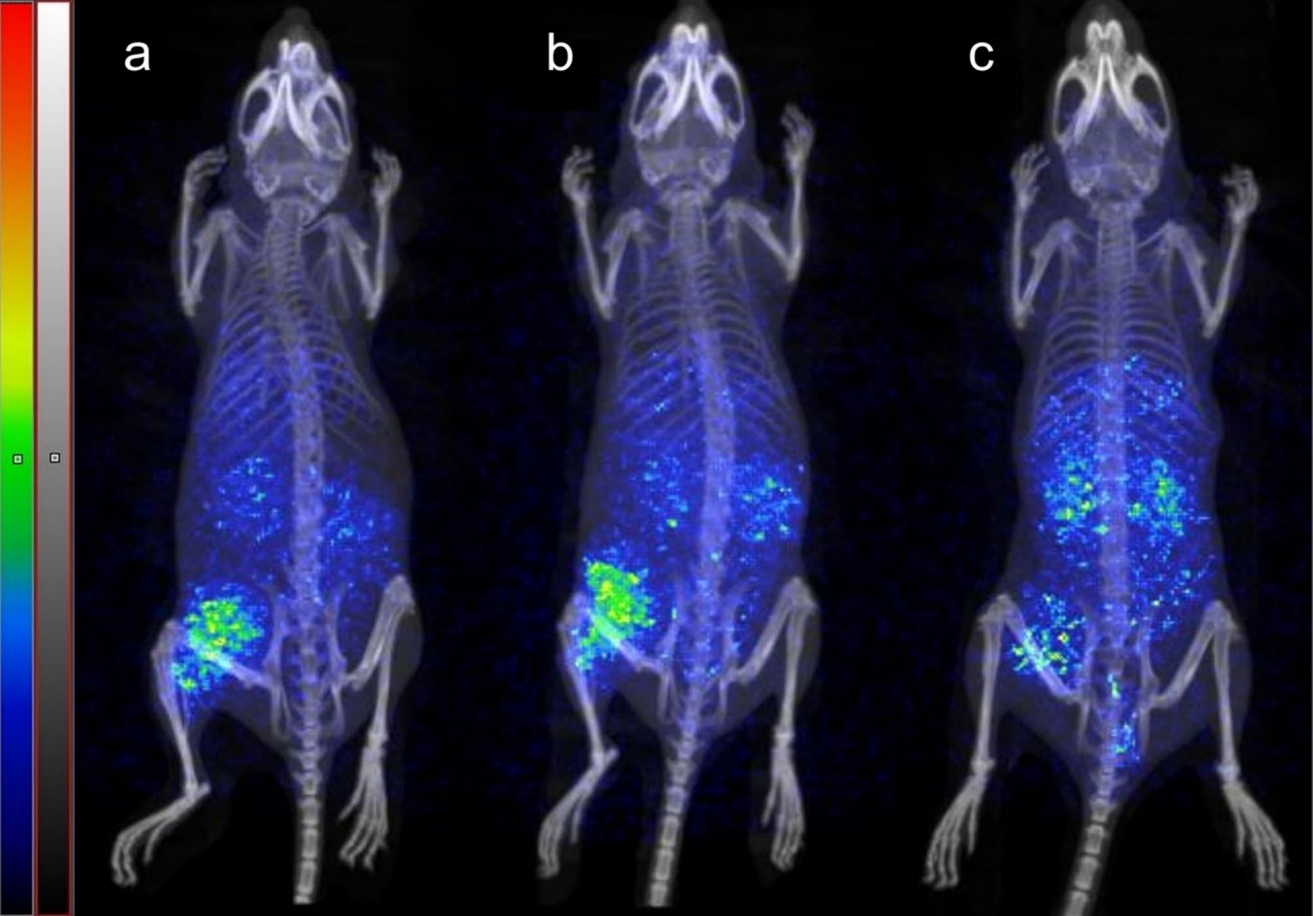
PET/CT scans of (**a**) [^68^Ga]Ga-NOTA-*o*ET-RM26, (**b**) [^68^Ga]Ga-NOTA-*o*MA-RM26 and (**c**) [^68^Ga]Ga-NOTA-*m*MA-RM26. Images are presented in maximal intensity of full body projections on the same scale (minimum: 0 kBq/mL, maximum: 240 kBq/mL)

Images of mice bearing PC-3 xenografts were acquired 2 h post injection (Fig. 4). The PET images were in accordance with the biodistribution data. All the radioligands demonstrated accumulation in GRPR-expressing PC-3 tumors, and *m*MA compound showed a lower uptake compared to the *o*ET and *o*MA analogs. The radioligands also had activity accumulation in abdominal tissues, kidneys, and liver. The *o*ET tracer demonstrated the lowest signal in the liver area on PET images, and the ^68^Ga-radiolabeled *o*ET and *o*MA peptides enable clear identification of the tumor sites.

## Discussion

In this study, we performed a comparison of binding specificity, cellular processing, and pharmacokinetics of ^68^Ga-labeled GRPR targeting peptides with conformationally restricted linkers joining a RM26-targeting motif and a NOTA chelator. The targeting domain of all bombesin analogues is based on a hydrophobic peptide sequence that is important for binding to GRPR [25, 26]. In contrast, the radiometal chelator is highly polar, and the spacer region linking these two functional domains provides an opportunity to tune and optimize radiotracer properties. The presence of short hydrophilic linkers, such as (PEG)_n_ chains, usually decreases hepatobiliary clearance while increasing renal clearance by balancing non-specific interactions [27, 28]. The inclusion of longer PEG spacers leads to undesired lower whole-body clearance kinetics [28]. Similarly, compounds with aliphatic linkers are cleared via biliary excretion pathways [29], which are slower and unfavorable for diagnostic imaging. However, the relationship between hydrophobicity, binding properties and pharmacokinetics is subtle, and peptides with moderately hydrophobic linkers have demonstrated successful pre-clinical and clinical results [25, 30]. For instance, the [^68^Ga]Ga-SB3 antagonist was found to be safe and effective in the imaging of primary prostate cancers with excellent imaging qualities [31]. [^68^Ga]Ga-NeoBOMB1 has shown promising PET imaging of GRPR expression in gastrointestinal stromal tumors in Phase I/IIa clinical studies [32]. The other promising GRPR antagonists with partially hydrophobic linkers include the TacsBOMB family [33] and RM1 with glycine-4-aminobenzoyl spacer [18]. In this study, the increased hydrophobicity of the aromatic turn motif was balanced by incorporation of an α-acetyl-lysine bridge in the spacer (Fig. 1).

It is known that the pharmacokinetic and pharmacodynamic properties of a radiotracer can be modified depending on the linker. Therefore, we prepared three GRPR-targeting analogues based on NOTA-PEG_2_-RM26 [22]. Here, we investigated how the linker between the NOTA chelator and the RM26 targeting motif influenced peptide interaction with the target both in vitro and in vivo. Each linker included one of the moderately hydrophobic and restricted turns: *o*-ethyltoluene (*o*ET ), *o*-methylanisole (*o*MA ), *m*-methylanisole (*m*MA) linked through an *α*-acetyl-lysine bridge to the chelator.

The introduction of *o*-ethyltoluene, *o*-methylanisole, or *m*-methylanisole based linkers did not greatly affect receptor binding, and the affinity of all three peptides was in the single-digit nanomolar range, similar to NOTA-PEG_2_-RM26. The *o*ET, which has an intermediate lipophilicity, had an IC_50_ of 3.4 nM, 8.5 times worse than for ^nat^Ga-NOTA-PEG_2_-RM26 (0.4 nM). In contrast, the *o*MA and *m*MA analogues had IC_50_ values of 1.3 nM and 0.27 nM, respectively. Therefore, incorporation of longer and rigid linkers did not affect the affinity of binding to a target, as follows from the comparison of PEG_2_ and *m*MA compounds. However, a direct comparison between the new peptides shows that both restricted turn incorporation and increased hydrophobicity lead to a decrease in binding affinity.

The cellular processing of ^68^Ga-labeled GRPR binding peptides was assessed over 3 h. Longer time points are not relevant since the half-life of ^68^Ga (67.7 min) enables the clinical imaging to be performed only shortly after compound injection. The structural modifications did not change their behavior from antagonists to agonists. All three newly designed compounds demonstrated low internalization kinetics with lower than 10% of the ligand found inside the cells after 3 h, a precondition for rapid clearance from healthy GRPR expressing organs, e.g., pancreas [17, 34]. Low internalization is expected for GRPR antagonists since no activation of the receptors occurs [34]. Antagonists with high affinity to GRPR are favorable for PET imaging due to strong receptor binding and low background by rapid washout from normal tissues [34].

The three newly designed peptides demonstrated a correlation between specific binding on PC-3 cells in vitro and in vivo binding to healthy tissues with overexpression of GRPR (pancreas, intestine, and stomach). The bombesin analogues bind to the IV extracellular domain of GRPR, which is identical in mice and humans [26]. Also, the human pancreas has the highest GRPR expression level across normal tissues [24]. The highest accumulation and binding were observed for the *o*MA peptide, which is the most lipophilic among the three new analogues.

It is interesting to compare the obtained results with the pharmacokinetics of the hydrophilic analog, [^68^Ga]Ga-NOTA-PEG_2_-RM26 [22]. A biodistribution study conducted at the same administered dose (40 pmol) and the same time point demonstrated faster clearance from the body than novel peptides. The comparison of tumor activity accumulation has shown a 2-fold increase for the novel peptides 2 h after injection [22]. Therefore, the presence of hydrophobic turns did not disrupt binding of the peptides to GRPR both in vitro and *in vivo.*

The blood concentrations of the newly designed peptides and the PEG_2_ analogue were similar. However, the PEG_2_ peptide showed significantly lower accumulation in the lungs (0.17 ± 0.03%IA/g), liver (1 ± 0.1%IA/g), and kidneys (1.9 ± 0.3%IA/g). Similarly, tissue uptake in GRPR-expressing regions was also reduced: tumor (5.4 ± 1%IA/g), pancreas (1.9 ± 0.1%IA/g), stomach (1.4 ± 0.2%IA/g), and small intestine (0.6 ± 0.1%IA/g) [22]. Therefore, tumor uptake was noticeably higher for all newly designed peptides than for [^68^Ga]Ga-NOTA-PEG_2_-RM26. However, the incorporation of more lipophilic and rigid linkers also resulted in tissue activity retention, likely due to off-target interactions. One potential off-target is the Low-Density Lipoprotein Receptor (LDLR), which is expressed in almost all tissues and binds lipophilic molecules present in plasma [35]. Moreover, this receptor is overexpressed in hepatocytes of the liver, which may explain the high signal observed in the gastrointestinal tract and liver, attributed to the involvement of the hepatobiliary excretion pathway in the elimination of activity.

The most common sites for prostate cancer metastasis in humans are the bones, liver, and lungs [36]. Pharmacokinetic analysis indicated that visualization of prostate cancer metastases is likely to be most effective using the *o*ET tracer, despite the increase in lipophilicity, as it demonstrated lower accumulation in the liver and lungs. Additionally, imaging of metastases in the pancreas, intestine, peritoneum, and regional paraaortic or peripancreatic lymph nodes will be complicated for all new analogues due to high background from gastrointestinal tissues in the abdominal region.

In clinical settings, primary prostate cancer can have GRPR overexpression at early stages of disease, while normal prostate and surrounding tissues do not express this receptor. Thus, the high tumor-to-tissue values clearly demonstrated that ^68^Ga-labeled novel peptides are promising candidates for further development of diagnostic tracers for GRPR-positive cancers.

It is interesting to compare the behavior of the two pairs of linkers from a structural point of view. The *o*MA and *m*MA linkers dispite being isomeric, have different lipophilicity, and differ in the relative orientation of the two functional domains around the aromatic core. On the other hand, the *o*ET and *o*MA are both turn motifs, that share the orientation around the phenyl ring but have quite close values in terms of lipophilicity (Table 1). The *o*MA linker peptide has higher binding *in vitro*, in cell specificity and cell internalization assays, and higher in vivo accumulation in pancreatic tissue, while the best IC_50_ value was observed for the *m*MA compound, which has the highest hydrophilicity among the new analogues.

From the in vivo data, all three compounds demonstrated equally efficient accumulation in GRPR expressing tumors. Both methylanisole derivatives (*o*MA and *m*MA) have higher accumulation in healthy tissues than *o*ET peptides, highlighting the effects of minor structural modifications on non-specific off-target interactions in lungs, liver, and spleen. Notably, the *m*MA peptide has lower pancreas accumulation than the *o*MA analogue, likely due to a faster washout from the tissue.

Finally, incorporating aromatic and conformationally restricted linkers into bombesin analogues opens new avenues for exploring branched peptides in radiopharmaceutical development. Modifications to the spacer had minimal impact on RM26’s binding affinity to GRPR, however, some effects were noted on off-target interactions. Notably, some radioligands exhibited improved tumor retention compared to analogues with a standard hydrophilic linear PEG_2_ linker. The synthetic route also allows for further diversification, including substitutions on the aromatic ring and the introduction of alternative aromatic systems within the spacer. This flexibility offers the potential to incorporate branched moieties into the peptide, aiming to further enhance pharmacokinetic and pharmacodynamic properties. For example, the addition of albumin-binding motifs can significantly prolong the blood circulation time of the compound, required for therapeutic applications [37]. Additionally, these branched modifications are well-suited to the synthesis of homodimers that can provide higher avidity of compound-GRPR interaction and thus, potentially improved properties [38].

## Conclusion

In this study, we designed a novel family of GRPR antagonists based on the RM26 targeting moiety, a NOTA chelator, and a series of spacers featuring conformationally restricted turns and extended motifs. Following radiolabeling with ⁶⁸Ga, the resulting radiopharmaceuticals exhibited clear antagonistic behavior and high specificity for GRPR. Biodistribution studies in mice bearing human xenografts, along with PET imaging, indicated that the compound with the intermediate lipophilicity, *o*ET, holds promise for further clinical evaluation. Although this compound showed slightly lower affinity compared to other peptides, its IC_50_ remained in the nanomolar range and is comparable to values observed in other preclinical and clinical candidates. Importantly, its rapid clearance from non-target tissues helped reduce background signal in abdominal organs, enhancing imaging quality. These findings highlight the potential of novel analogue design in developing next-generation GRPR-targeted radiopharmaceuticals, paving the way for more precise and effective PET imaging of prostate cancer.

## Supplementary Information

Below is the link to the electronic supplementary material.

Supplementary Material 1

## Data Availability

The data generated during the current study are available from the corresponding author upon reasonable request.

## Abbreviations

GRPR: Gastrin releasing peptide receptor
GI: Gastrointestinal
mMA − 2: (3-(((((9 H-fluoren-9-yl)methoxy)carbonyl)amino)methyl)phenoxy)acetic acid
NOTA − 1,4,7: Triazacyclononane-1,4,7-triacetic acid
oET − 2: (2-(2-((((9 H-fluoren-9-yl)methoxy)carbonyl)amino)ethyl)phenyl)acetic acid
oMA − 2: (2-(((((9 H-fluoren-9-yl)methoxy)carbonyl)amino)methyl)phenoxy)acetic acid
PET: Positron emission tomography
SPECT: Single-photon emission computed tomography
