# Supplementary material for: A preclinical study on the influence of linkers in [68Ga]Ga-NOTA-X-RM26 radiotracers for PET imaging of GRPR expression
Source: EJNMMI Res. 2025 Aug 7;15:104. doi: 10.1186/s13550-025-01301-y (PMC12332157; doi:10.1186/s13550-025-01301-y)
Supplement: Supplementary file 1 — Supplementary Material 1 [file 13550_2025_1301_MOESM1_ESM.docx]

**Supplementary Information**

**A preclinical study on the influence of linkers in [^68^Ga]Ga-NOTA-X-RM26 radiotracers for PET imaging of GRPR expression**

Esther Olaniran Håkansson^1^*, Ivan V. Zelepukin^1,2^*, Karim Obeid^1^, Athanasios Bitzios^1^, Ekaterina Bezverkhniaia^1^, Amulya Sunkara^1^, Ulrika Rosenström^1^, Anna Orlova^1,3^, Luke R. Odell^1#^, Panagiotis Kanellopoulos^1#&^

^1^Department of Medicinal Chemistry, Uppsala University, 751 83 Uppsala, Sweden

^2^ Department of Immunology, Genetics and Pathology, Uppsala University, 751 83 Uppsala, Sweden

^3^ Science for Life Laboratory, Uppsala University, 752 37 Uppsala, Sweden

* These authors contributed equally

^#^ These authors contributed equally

& Corresponding author: Panagiotis Kanellopoulos, Panagiotis.kanellopoulos@ilk.uu.se

**Structure of NOTA-*o*ET-RM26**

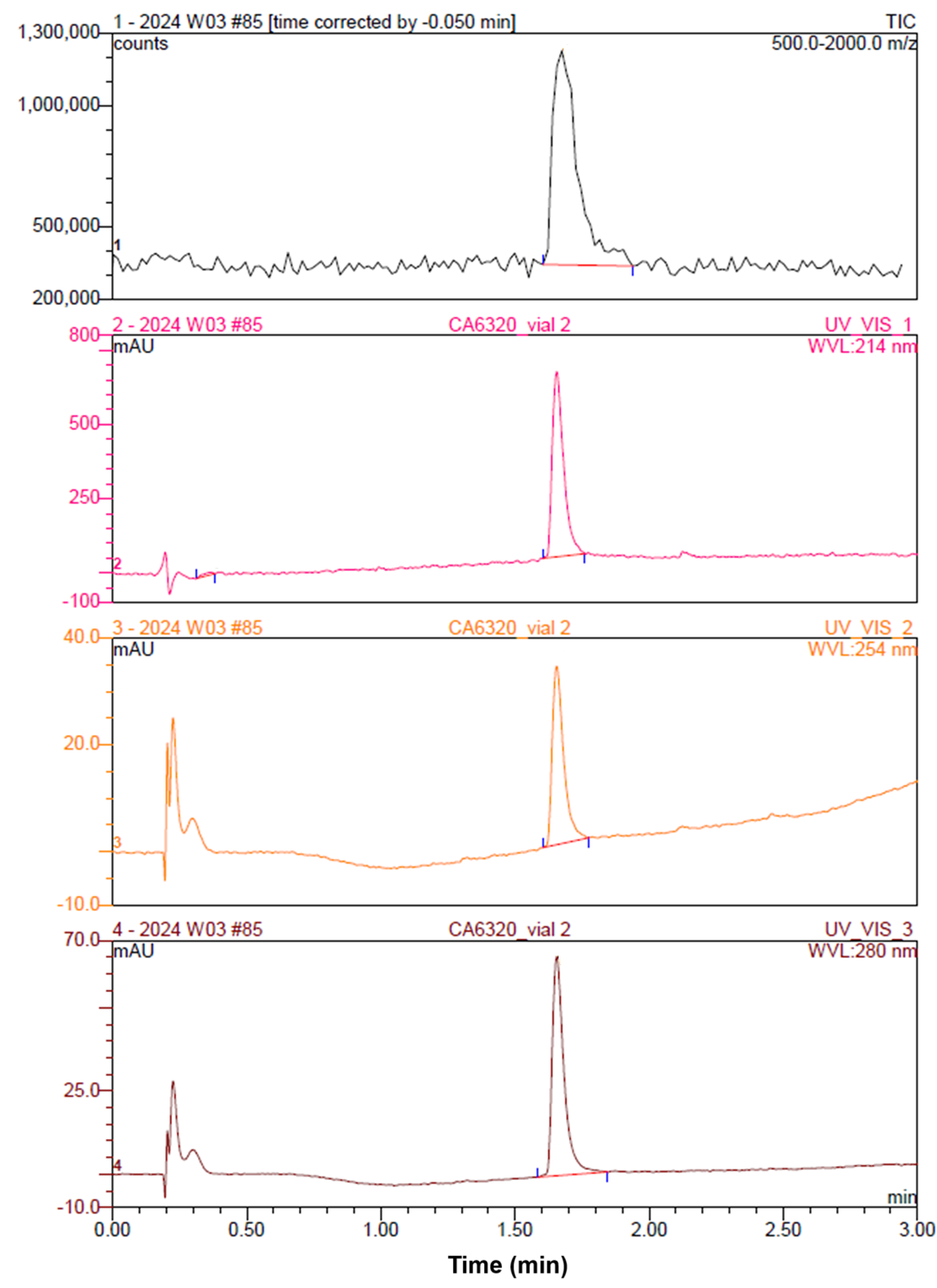


**Figure S1.** Analytical RP-HPLC of NOTA-oET-RM26 on C18 column, acetonitrile gradient 5-100% of 0.05% formic acid in acetonitrile/water for 3 min. Top to bottom: total ionization chromatogram (TIC), UV detection at 214, 254, and 280 nm.


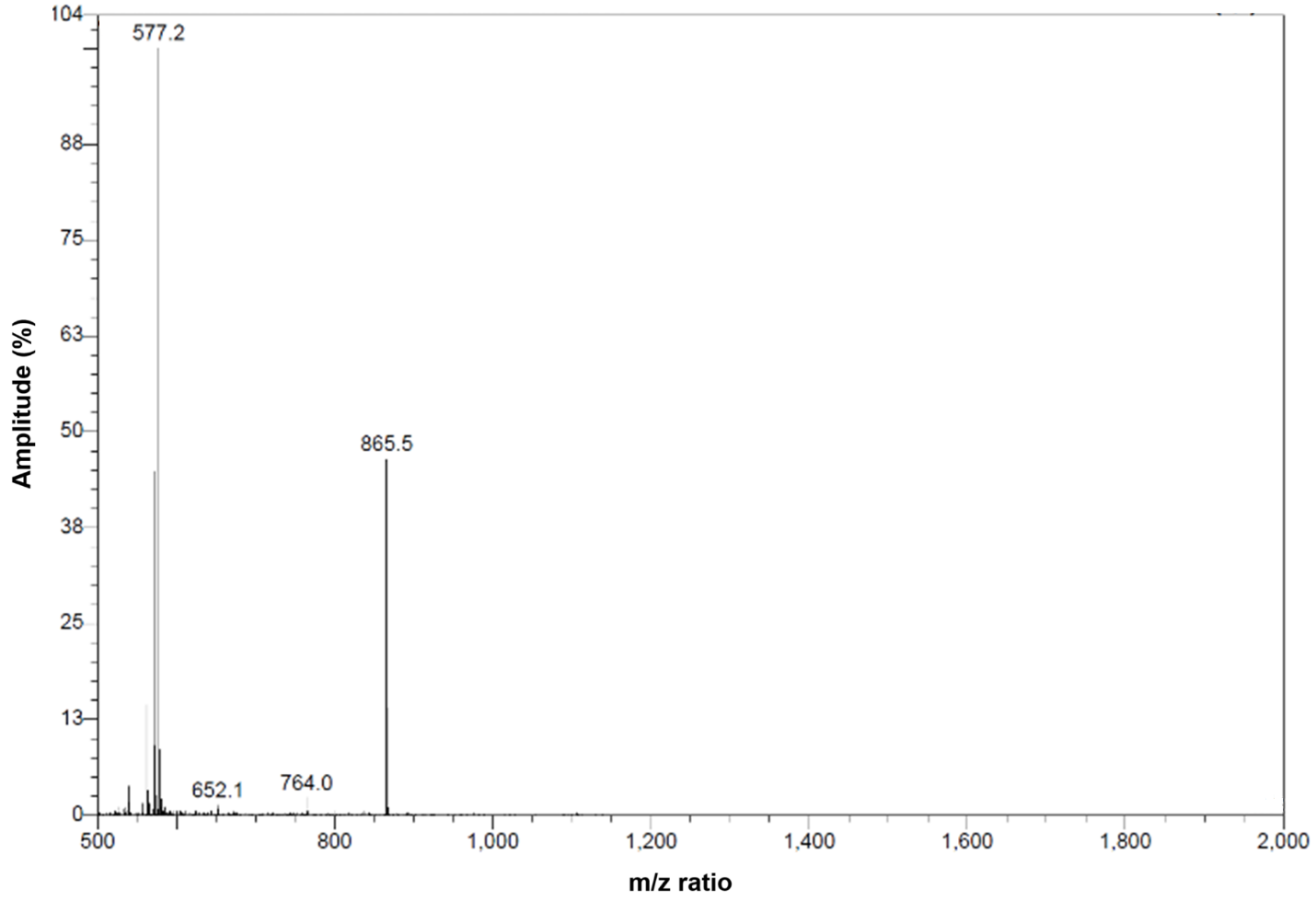


**Figure S2.** Low-resolution mass spectrum of NOTA-oET-RM26. Calculated [M+2H]^2+^ and [M+3H]^3+^: 866.0 and 577.7, respectively. Observed [M+2H]^2+^ and [M+3H]^3+^: 865.5 and 577.2, respectively.

**Figure S3**. High-resolution mass spectrum (HRMS) of NOTA-oET-RM26.

**Structure of NOTA-*o*MA-RM26**

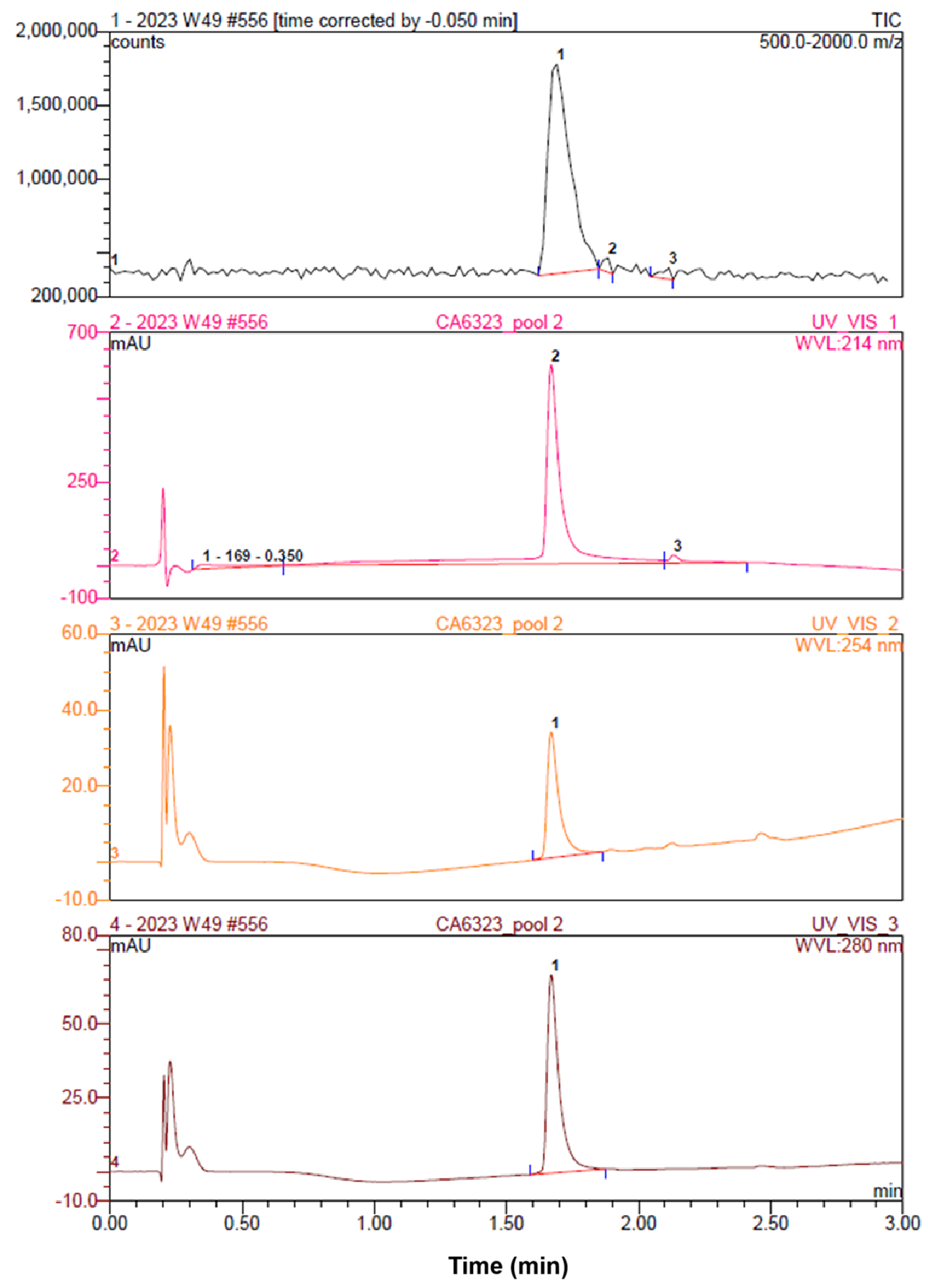


**Figure S4.** Analytical RP-HPLC of NOTA-oMA-RM26 on C18 column, acetonitrile gradient 5-100% of 0.05% formic acid in acetonitrile/water for 3 min. Top to bottom: total ionization chromatogram (TIC), UV detection at 214, 254, and 280 nm.

**
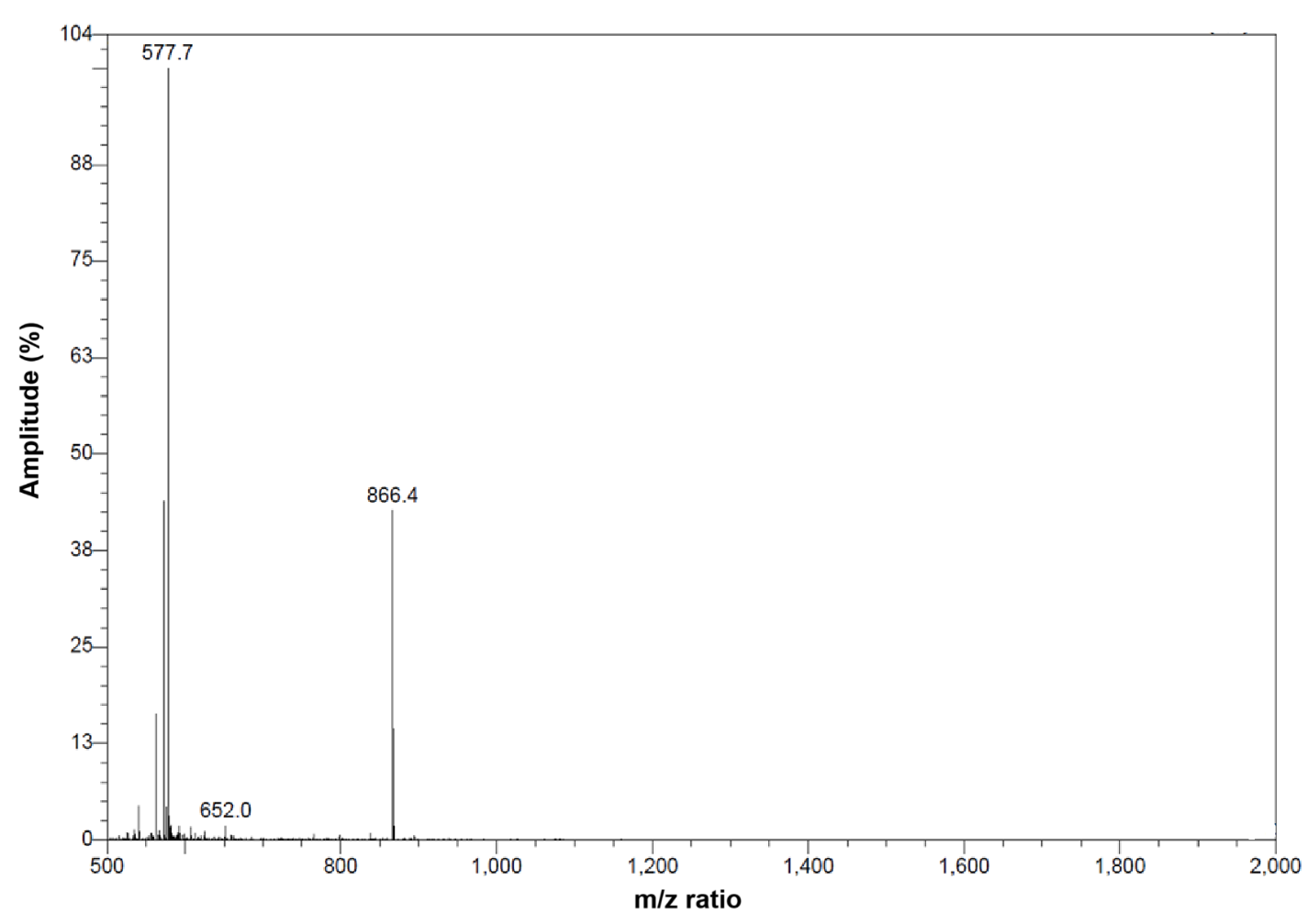
Figure S5.** Low-resolution mass spectrum of NOTA-oMA-RM26. Calculated [M+2H]^2+^ and [M+3H]^3+^: 867.0 and 578.4, respectively. Observed [M+2H]^2+^ and [M+3H]^3+^: 866.4 and 577.7, respectively.

**Figure S6**. High-resolution mass spectrum (HRMS) of NOTA-oMA-RM26.

**Structure of NOTA-*m*MA-RM26**

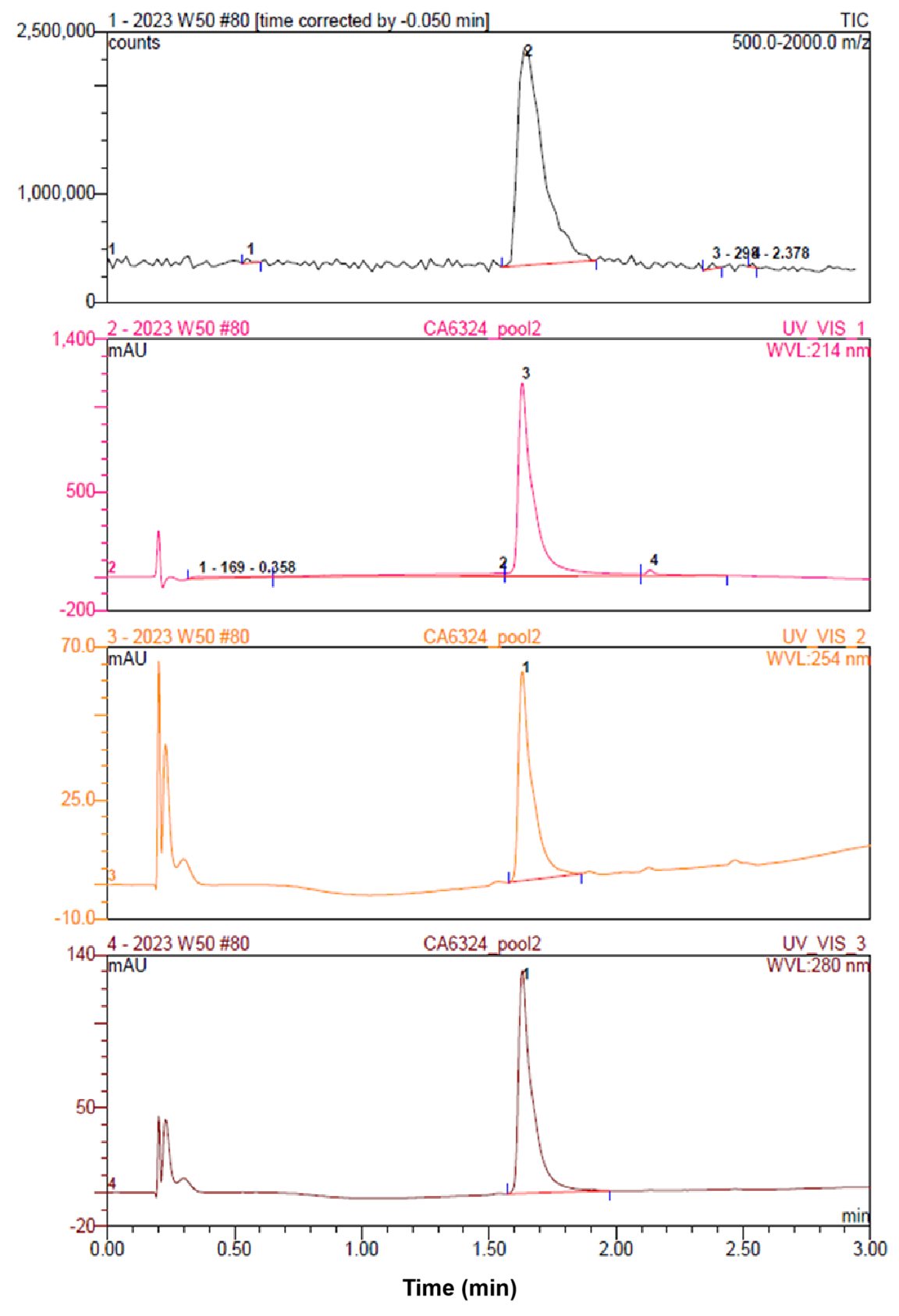


**Figure S7.** Analytical RP-HPLC of NOTA-mMA-RM26 on C18 column, acetonitrile gradient 5-100% of 0.05% formic acid in acetonitrile/water for 3 min. Top to bottom: total ionization chromatogram (TIC), UV detection at 214, 254, and 280 nm.


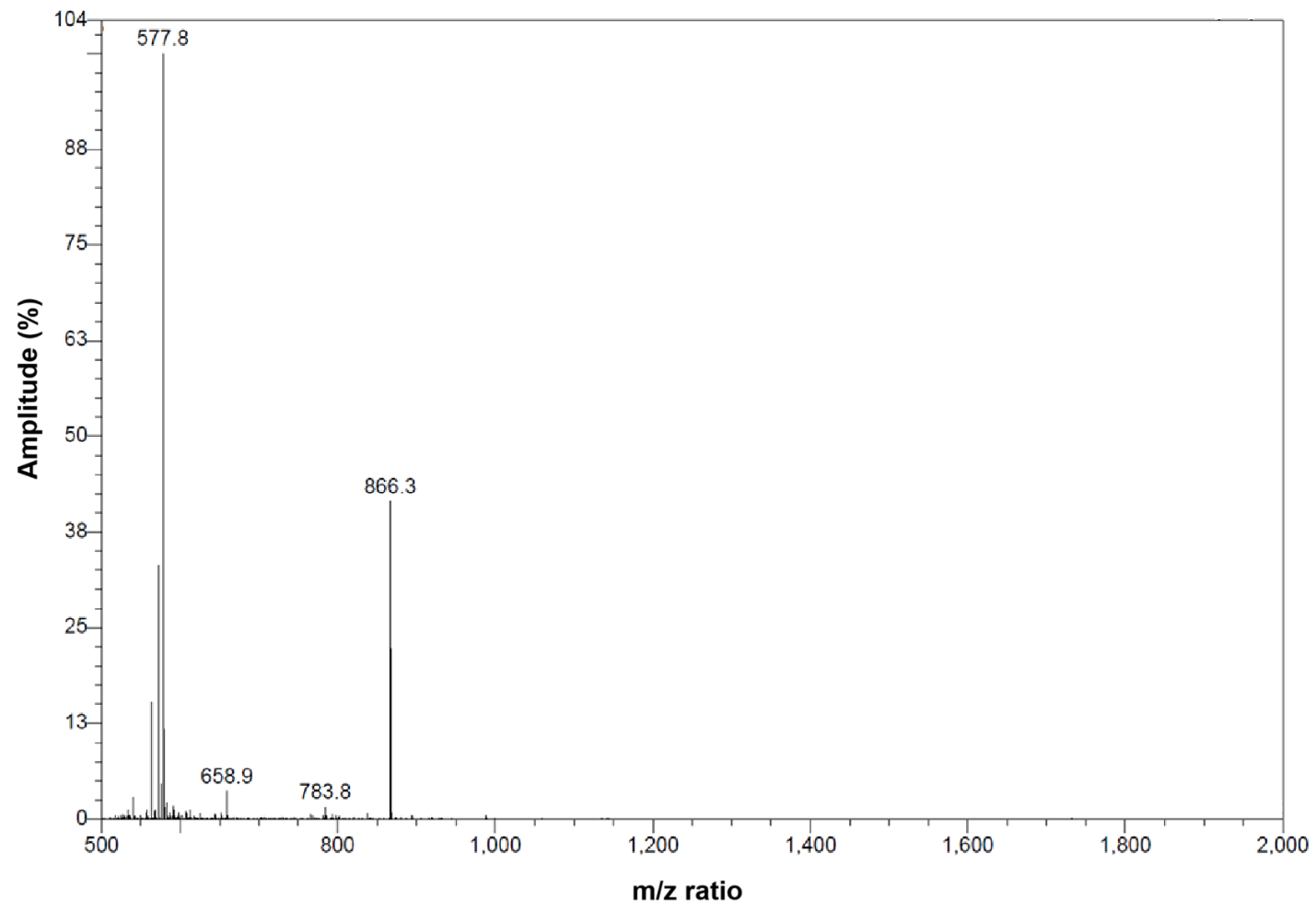


**Figure S8.** Low-resolution mass spectrum of NOTA-mMA-RM26. Calculated [M+2H]^2+^ and [M+3H]^3+^: 867.0 and 578.4, respectively. Observed [M+2H]^2+^ and [M+3H]^3+^: 866.3 and 577.8, respectively.

**Figure S9**. High-resolution mass spectrum (HRMS) of NOTA-mMA-RM26.


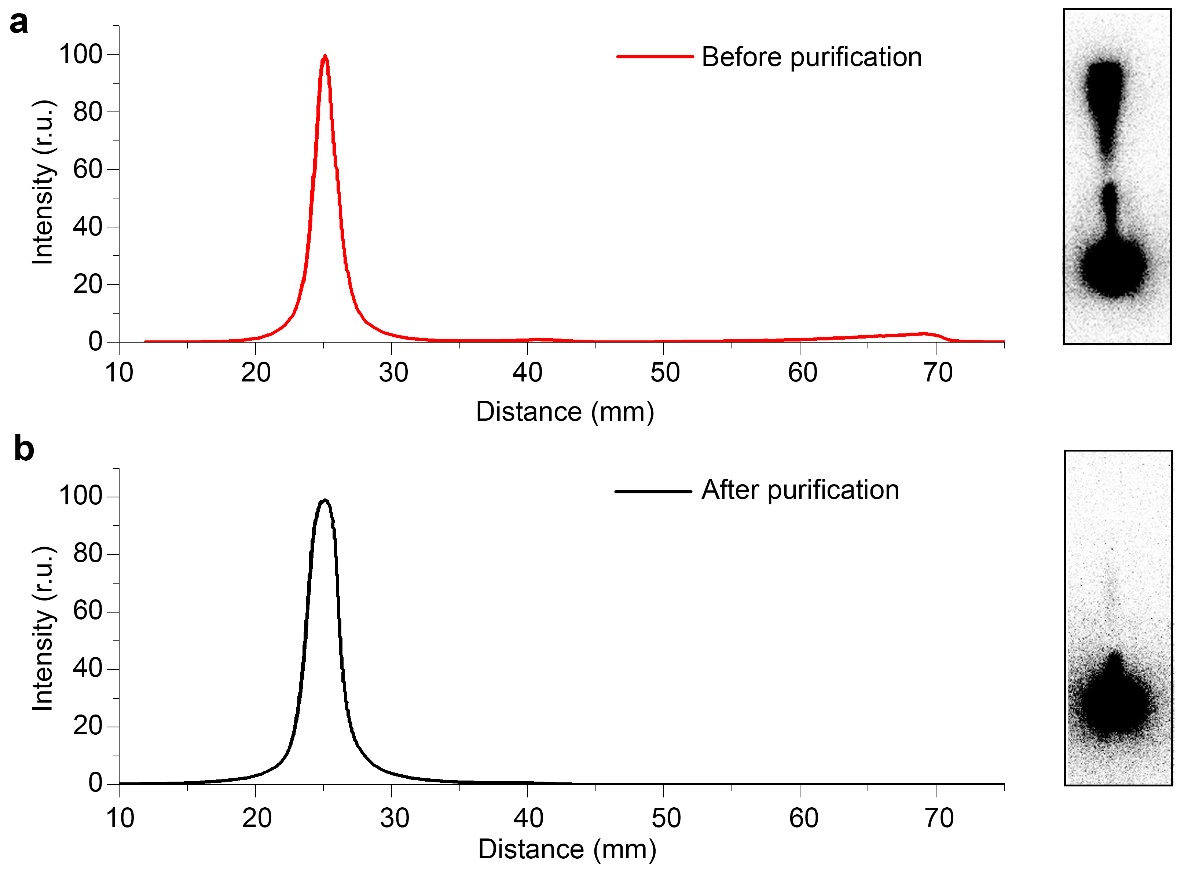


**Figure S10.** **(a,b)** Representative iTLC profile of [^68^Ga]Ga-NOTA-oET-RM26 peptide before **(a)** and after **(b)** purification via solid-phase extraction. Phosphor images of corresponding strips are shown on the right.


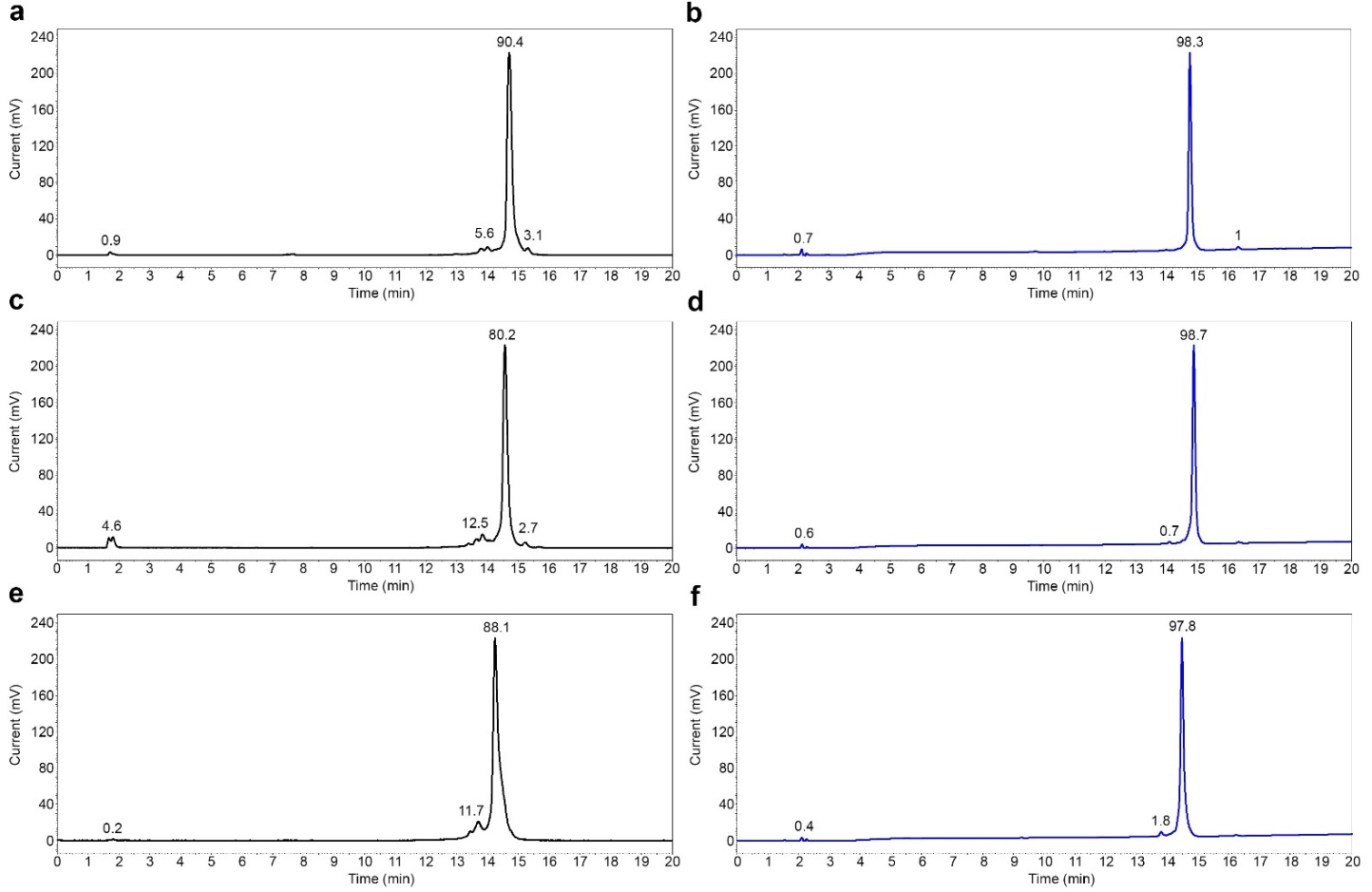


**Figure S11.** **(a,b)** Reverse-phase HPLC profile of NOTA-oET-RM26 **(a,b)**, NOTA-oMA-RM26 **(c,d)**, NOTA-mMA-RM26 **(e,f)** peptides. HPLC profiles are shown for ^68^Ga-labeled peptides in radio-channel **(a,c,e)** and for non-labeled peptides in UV-channel **(b,d,f)**. Areas under the identified peaks (%) are shown on the graphs. No decay correction was done.


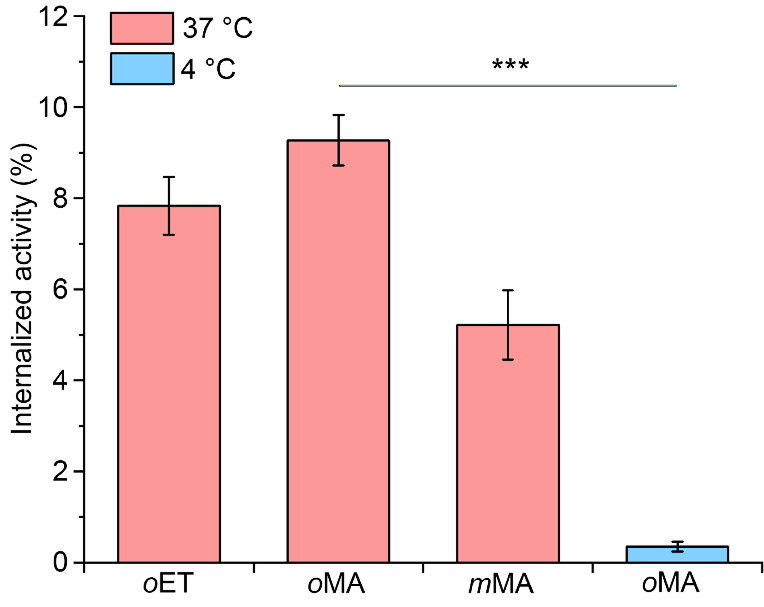


**Figure S12.** Amounts of ^68^Ga-labeled RM26 peptides internalized by PC-3 cells after co-incubation for 3 h at 37 °C or at 4 °C. Data are presented as mean ± SD. *** - P < 0.001, Welch’s t-test..

**Table S1.** Biodistribution data for [^68^Ga]Ga-NOTA-oET-RM26 in PC-3 xenograft bearing BALB/c nu/nu mice. Total activity was calculated as sum of the measured activities. Data are presented as mean ± SD. P value calculated using Welch’s t-test.

|  | **Radionuclide concentration (% IA/g)** | |  |
| --- | --- | --- | --- |
| **Tissue** | **2 h** | **Blockade, 2 h** | **P value** |
| **Blood** | 0.54 ± 0.01 | 0.31 ± 0.04 | 0.001 |
| **Lungs** | 0.45 ± 0.27 | 0.37 ± 0.02 | 0.58 |
| **Liver** | 1.9 ± 0.2 | 1.3 ± 0.1 | 0.004 |
| **Spleen** | 0.46 ± 0.06 | 0.28 ± 0.07 | 0.009 |
| **Pancreas** | 11.7 ± 2.4 | 1.2 ± 0.2 | 0.003 |
| **Stomach** | 2.6 ± 0.6 | 0.55 ± 0.19 | 0.003 |
| **Small Intestines** | 2.4 ± 0.4 | 0.33 ± 0.24 | < 0.001 |
| **Kidneys** | 5.9 ± 0.6 | 3.3 ± 2.3 | 0.11 |
| **Tumor** | 11.2 ± 3.4 | 1.3 ± 0.2 | 0.01 |
| **Muscle** | 0.15 ± 0.01 | 0.15 ± 0.13 | 0.98 |
| **Bone** | 0.44 ± 0.04 | 0.33 ± 0.07 | 0.03 |
|  | **Radionuclide quantity (% IA)** | |  |
| **Gastrointestinal tract** | 5.9 ± 0.5 | 3.5 ± 0.7 | 0.007 |
| **Carcass** | 4.1 ± 1.1 | 3.1 ± 1.8 | 0.002 |
| **Total** | 16.3 ± 1.6 | 8.5 ± 3.0 | 0.46 |

**Table S2.** Biodistribution data for [^68^Ga]Ga-NOTA-oMA-RM26 in PC-3 xenograft bearing BALB/c nu/nu mice. Total activity was calculated as sum of the measured activities. Data are presented as mean ± SD. P value calculated using Welch’s t-test.

|  | **Radionuclide concentration (% IA/g)** | |  |
| --- | --- | --- | --- |
| **Tissue** | **2 h** | **Blockade, 2 h** | **P value** |
| **Blood** | 0.47 ± 0.17 | 0.22 ± 0.08 | 0.054 |
| **Lungs** | 5.7 ± 1.5 | 3.5 ± 2.2 | 0.15 |
| **Liver** | 3.5 ± 0.5 | 1.4 ± 0.4 | < 0.001 |
| **Spleen** | 1.2 ± 0.3 | 0.37 ± 0.08 | 0.012 |
| **Pancreas** | 21.0 ± 5.2 | 2.5 ± 0.6 | 0.005 |
| **Stomach** | 3.5 ± 0.7 | 1.1 ± 0.7 | 0.003 |
| **Small Intestines** | 3.4 ± 1.3 | 0.61 ± 0.13 | 0.024 |
| **Kidneys** | 6.3 ± 0.9 | 6.2 ± 1.3 | 0.87 |
| **Tumor** | 10.2 ± 2.7 | 1.0 ± 0.4 | 0.006 |
| **Muscle** | 0.16 ± 0.05 | 0.21 ± 0.09 | 0.44 |
| **Bone** | 0.34 ± 0.05 | 0.23 ± 0.03 | 0.014 |
|  | **Radionuclide quantity (% IA)** | |  |
| **Gastrointestinal tract** | 6.5 ± 0.7 | 4.1 ± 2.1 | 0.081 |
| **Carcass** | 2.9 ± 0.4 | 5.9 ± 2.7 | 0.099 |
| **Total** | 19.8 ± 2.6 | 15.7 ± 2.9 | 0.11 |

**Table S3.** Biodistribution data for [^68^Ga]Ga-NOTA-mMA-RM26 in PC-3 xenograft bearing BALB/c nu/nu mice. Total activity was calculated as sum of the measured activities. Data are presented as mean ± SD. P value calculated using Welch’s t-test.

|  | **Radionuclide concentration (% IA/g)** | |  |
| --- | --- | --- | --- |
| **Tissue** | **2 h** | **Blockade, 2 h** | **P value** |
| **Blood** | 0.39 ± 0.08 | 0.42 ± 0.21 | 0.84 |
| **Lungs** | 7.1 ± 4.2 | 3.4 ± 0.5 | 0.18 |
| **Liver** | 3.7 ± 1.4 | 2.2 ± 0.5 | 0.12 |
| **Spleen** | 1.1 ± 0.4 | 1.1 ± 0.8 | 0.95 |
| **Pancreas** | 10.5 ± 3.8 | 1.7 ± 0.9 | 0.016 |
| **Stomach** | 2.5 ± 0.6 | 0.77 ± 0.51 | 0.012 |
| **Small Intestines** | 1.7 ± 0.7 | 0.91 ± 0.48 | 0.12 |
| **Kidneys** | 8.4 ± 1.4 | 7.6 ± 0.7 | 0.4 |
| **Tumor** | 12.3 ± 2.8 | 1.8 ± 0.2 | 0.005 |
| **Muscle** | 0.12 ± 0.02 | 0.12 ± 0.05 | 0.94 |
| **Bone** | 0.28 ± 0.06 | 0.25 ± 0.03 | 0.5 |
|  | **Radionuclide quantity (% IA)** | |  |
| **Gastrointestinal tract** | 4.0 ± 0.4 | 3.1 ± 1.7 | 0.1 |
| **Carcass** | 2.8 ± 0.4 | 4.6 ± 0.7 | 0.45 |
| **Total** | 16.5 ± 3.1 | 12.6 ± 2.0 | 0.029 |
